# Double-Versus Triple-Potential Well Energy Harvesters: Dynamics and Power Output

**DOI:** 10.3390/s23042185

**Published:** 2023-02-15

**Authors:** Jerzy Margielewicz, Damian Gąska, Jacek Caban, Grzegorz Litak, Agnieszka Dudziak, Xiaoqing Ma, Shengxi Zhou

**Affiliations:** 1 Faculty of Transport and Aviation Engineering, Silesian University of Technology, Krasińskiego 8, 40-019 Katowice, Poland; 2 Faculty of Mechanical Engineering, Lublin University of Technology, Nadbystrzycka 36, 20-618 Lublin, Poland; 3 Faculty of Production Engineering, University of Life Sciences in Lublin, Głęboka 28, 20-612 Lublin, Poland; 4 School of Aeronautics, Northwestern Polytechnical University, Xi’an 710072, China

**Keywords:** Lyapunov exponent, bifurcations, multiple solutions diagram, optimization

## Abstract

The basic types of multi-stable energy harvesters are bistable energy harvesting systems (BEH) and tristable energy harvesting systems (TEH). The present investigations focus on the analysis of BEH and TEH systems, where the corresponding depth of the potential well and the width of their characteristics are the same. The efficiency of energy harvesting for TEH and BEH systems assuming similar potential parameters is provided. Providing such parameters allows for reliable formulation of conclusions about the efficiency in both types of systems. These energy harvesting systems are based on permanent magnets and a cantilever beam designed to obtain energy from vibrations. Starting from the bond graphs, we derived the nonlinear equations of motion. Then, we followed the bifurcations along the increasing frequency for both configurations. To identify the character of particular solutions, we estimated their corresponding phase portraits, Poincare sections, and Lyapunov exponents. The selected solutions are associated with their voltage output. The results in this numerical study clearly show that the bistable potential is more efficient for energy harvesting provided the corresponding excitation amplitude is large enough. However, the tristable potential could work better in the limits of low-level and low-frequency excitations.

## 1. Introduction

Energy harvesting is a method developed since the beginning of the 21st century to obtain electricity from ambient sources, such as vibration [1,2], rotation [3,4] air flow [5,6], and temperature changes [7]. Various aspects of energy harvesting with respect to the vibration ambient sources are discussed and summarized in reviews [8,9,10]. The most popular application is the conversion of mechanical energy into electrical energy, widely developed in the scientific literature [8,11]. Such systems are usually based on a flexible cantilever beam [12,13] with a piezoelectric transducer attached to it. Vibrations of the forcing object cause variable deformations of the beam and the transducer itself, and this allows generation of electricity. The obtained amounts of energy are small, but sufficient to power the sensors and send the obtained information wirelessly [4]. Such systems can be successfully used in hard-to-reach places due to the lack of the need to replace the battery or the supply of main power.

The main challenge for scientists is to maximize the power output of the particular harvester design [14]. Note that the first applications of energy harvesters are based on linear systems tuned to the frequency of the excitation source. They allow effective energy acquisition near the resonant frequency of the systems. Therefore, their design and application are limited to a specific source of excitation.

One of the intensively studied systems for converting mechanical energy into electricity is based on nonlinear solutions to harvest energy in a wider frequency range for broadband energy harvesting [11,15]. Nonlinearities are introduced by using permanent magnets on the beam tip and the housing of the energy harvester. Another design solution is the introduction of spring elements [16] or a possible buckling design [17]. The characteristic features of such solutions have a wide and effective range of application in relation to linear systems, but also cause the possibility of coexisting solutions’ occurrence [18,19]. Recently, solutions including tunable dynamics energy harvesting systems [20], higher frequency systems [21], multi-array systems [22], and self-adaptive systems [23] were also explored.

The basic type of multi-stable energy harvesters is bistable energy harvesting systems (BEH) with one unstable and two stable equilibrium positions (with two potential wells). In this case, we distinguish single-beam systems based on nonlinear magnetic interaction, for example, in [24], and others based on multiple beam structures [25]. One of the interesting solutions was presented in [26], where the results of bending and torsion were considered simultaneously in the working condition. Other design solutions were proposed in [27,28]. An extension of BEHs is tristable energy harvesting systems (TEH), with one more potential well. Such solutions were widely scientifically researched, both in the field of symmetric and asymmetric potential wells [29]. The main design assumption is similar and consists in adding one permanent magnet in the construction. This results in an extension of the potential characteristics and a wider range of applications of the systems. There are many examples of TEH systems, related to their efficiency, dynamics, and coexisting solutions in the scientific literature [12,13,16,30,31,32,33].

The motivation for the research is that the results that are presented in this study are from a comparative analysis of various solutions for BEH and TEH which were presented in previous scientific works. It seems interesting to compare the efficiency of energy harvesting for such systems assuming similar potential parameters, which has not been carried out so far. Our research focused on the analysis of BEH and TEH, in which the depth of the potential well and the width of the characteristics are the same. Ensuring such parameters allows for a reliable formulation of conclusions about the efficiency in both types of systems. In this paper, we present a comparison of systems based on permanent magnets and a cantilever beam designed to harvest energy from vibrations.

## 2. Formulation of the Mathematical Model

**Figure 1:**
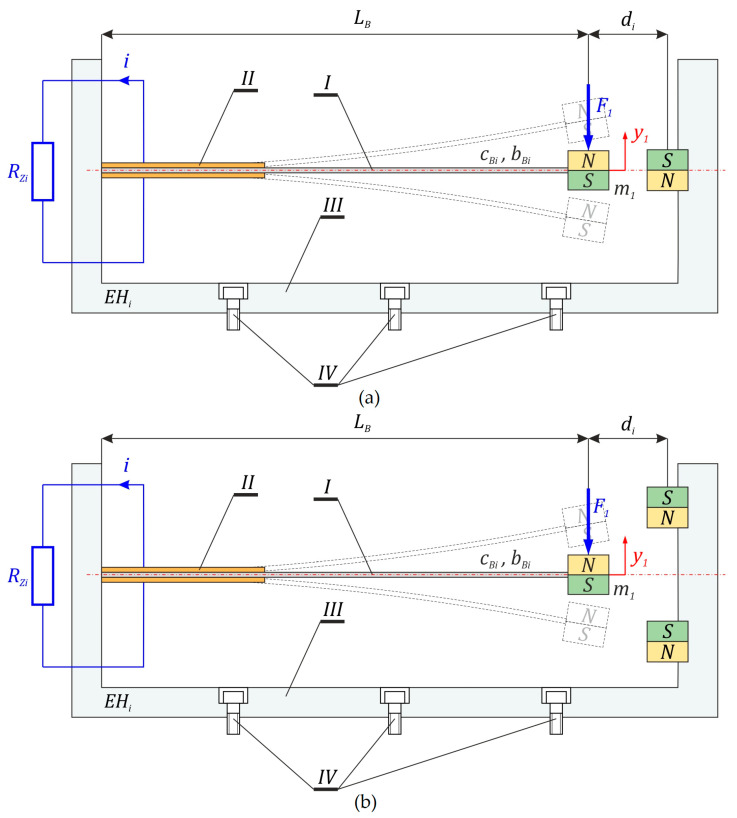
Analyzed design of energy harvesting systems with the magneto-piezo-elastic beam. Considered potentials are presented in (**a**) two-well and (**b**) three-well designs, respectively. The additional vertical arrow symbolizes the excitation contact force, *F*_1_, acting on the beam.

The subject of the model tests is energy harvesting systems whose potential is set by means of permanent magnets mounted in a rigid, non-deformable frame *III* (Figure 1). The considered structural solutions consist of a flexible cantilever beam *I*, which is clamped in a rigid frame *III*. This frame is fixed, using *IV* screws, to a mechanically vibrating object from which energy is recovered. On the flat surfaces of the flexible beam, piezoelectric elements *II* are attached to the suitable electrodes. During elastic deformations, the piezoelectric elements induce an electric charge on the electrodes.

**Figure 2:**
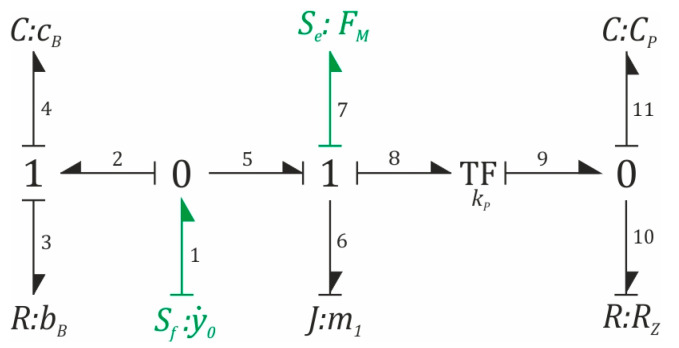
A graph of bonds representing the dynamics of the tested design solutions of energy harvesting systems.

The differential equations of motion reflecting the dynamics of the tested system can be derived by various methods. In our research, the equations of motion are derived by the bond graph method proposed by Paynter [34] and developed in the work of Karnopp et al. [35]. Each edge of the graph of bonds is represented by two variables: effort and flow, as a result of which the edges of the graph graphically depict the flow of power in the dynamic system under examination. In the method originally proposed by Paynter [34], the elements storing kinetic and potential energy are assigned integral causality. This causality is preferred because the bond graph method is intended to be a numerical tool that would allow computer simulations to be performed without explicit knowledge of the mathematical model. Moreover, from the point of view of numerical methods, it is much easier (without introducing additional errors to the experiment) to carry out the procedure of numerical integration. There are many computer applications that enable carrying out numerical calculations based on the bond graph method, the most popular being run in the Windows environment, with Mathematica and MATLAB: Windows 20sim [36], Mathematica Bond Graph Talk [37], BondLab [38], and newer ones. An undoubted advantage of the bond graph method is the possibility of modeling dynamic systems of various technical nature, i.e., electromechanical, hydromechanical, and electro-hydromechanical. Figure 2 shows the generalized structure of the bond graph, representing the dynamics of the formulated phenomenological models of the analyzed energy harvesting systems.

The formed graph of bonds consists of eleven edges and additional sources of effort and flow variables. The source of the effort variable (edge no. 7) models the impact of the magnetic field on the free end of the flexible cantilever beam *I*. The source of the flow variable (edge no. 1) represents the kinematic excitation describing the mechanical vibrations of the object from which the energy is recovered. During the automatic generation of the equations of motion, the elements storing kinetic and potential energy are assigned integral causality, as a result of which the graph can be directly adapted to computer simulations carried out in one of the aforementioned computer programs (Figure 2). In the case of the results of numerical calculations included in this work, a virtual mathematical model, saved in the form of connections in a graph and cause-and-effect relationships occurring in it, is of little help. For this reason, its explicit representation is still present.

Please note that in the case of one-line nodes, only one of the incident edges can be “open”. If all edges are closed, then there is a causality conflict. However, for null nodes, a causality conflict occurs when more than one of the edges’ incidents to a node is closed. At this point, it is worth noting that the causality analysis of the edges of the bond graph is one of the verification criteria of the formulated mathematical model. If there is a conflict in the graph, it is necessary to make a decision regarding the modification of the phenomenological model, which involves redefining the model assumptions.

**Figure 3:**
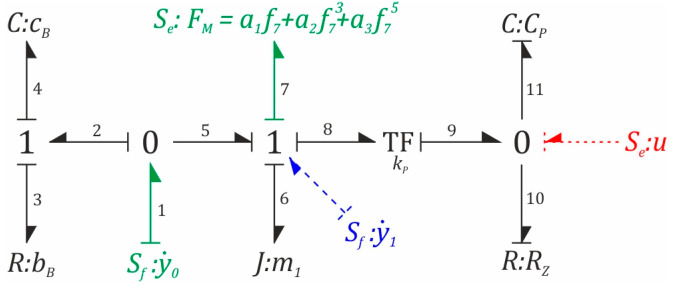
A Lagrange bond graph, with causality conflicts intentionally introduced.

Due to the fact that one of the secondary goals of this work is to approximate the method of bond graphs in the context of conducting model tests of energy harvesting systems, the differential equations of motion were derived using a modified method, in which the elements storing kinetic energy (elements in the graph marked with the letter *J*) were assigned differential causality (Figure 3). As a result of this approach, we obtained the so-called Lagrange bond graph, which is the formal basis for the derivation of the mathematical model in the form of a system of second-order differential equations. In the structure of a Lagrange bond graph, there is always a causality conflict that is deliberately initiated in order to enforce differential causality. The emergence of a causality conflict forces one node to hook additional fictitious edges of the source of the flow-type variable. Their introduction unambiguously defines the generalized coordinates of the graph. These edges in the bond graph (Figure 3) are highlighted by dashed lines in blue. However, these additional edges of the flow variable sources are not numbered. In the method of graphs of constraints, the flow sources are always incidental to the zero nodes, while the sources of the effort and effort variables are attached to the one node. Deriving the equations of motion from the Lagrange bond graph forces the introduction of additional fictitious sources, and they are connected to the nodes in the opposite way to the method originally proposed by Paynter [34]. Colored dashed lines highlight edges representing fictitious sources.

On the basis of the graph (Figure 3), the cause-and-effect relationships, corresponding to the one node in which the edge effort variables add up, while the flow variables are equal, were first recorded. In addition, the convention was adopted that if an edge “exits” from a unitary node, it is written with a “+” sign, otherwise the effort variable is marked with a “-” sign. Since each (numbered) edge is assigned two variables: effort and flow, therefore, the listed dependencies are grouped into two sets of equations:

(1) $$\begin{array}{cc} \left\{\begin{array}{l} e_{6}+e_{7}+e_{8}-e_{5}=0,\\ e_{3}+e_{4}-e_{2}=0, \end{array}\right. & \left\{\begin{array}{l} f_{6}=f_{7}=f_{8}=f_{5}={\dot{y}}_{1},\\ f_{3}=f_{4}=f_{2}, \end{array}\right. \end{array}$$

These equations, and in particular the first one, corresponding to the system of effort variables, describe the dynamics of the mechanical subsystem. The second equation, on the other hand, defines the forces induced in the flexible cantilever beam. In the zero nodes, the situation is reversed, the effort variables are equal, and the flow variables are subject to the law of superposition. As is the case for one node, two systems of equations are also written for the zero nodes of the graph. At the same time, the first equations correspond to the mechanical subsystem, and the second ones model the dynamics of the piezoelectric patch.

(2) $$\begin{array}{cc} \left\{\begin{array}{l} f_{2}+f_{5}-f_{1}=0\to f_{2}={\dot{y}}_{0}-{\dot{y}}_{1},\\ f_{10}+f_{11}-f_{9}=0, \end{array}\right. & \left\{\begin{array}{l} e_{1}=e_{2}=e_{5},\\ e_{9}=e_{10}=e_{11}=u. \end{array}\right. \end{array}$$

The relations between the variables describing the edges of the bond graph, the elements that store kinetic, potential, and electric energy, and the data responsible for its dissipation, are shown in Equation (3). At this point, it is worth mentioning that the presented dependencies were determined by the causality of the edges connecting these elements:

(3) $$\begin{array}{c} \begin{array}{cc} e_{6}=m_{1}\frac{df_{6}}{dt}=m_{1}\frac{d{\dot{y}}_{1}}{dt}, & e_{3}=b_{B}f_{3}=b_{B}\left({\dot{y}}_{0}-{\dot{y}}_{1}\right), \end{array}\\ e_{4}=c_{B}\int f_{4}dt=c_{B}\int \left({\dot{y}}_{0}-{\dot{y}}_{1}\right)dt,\\ e_{7}=a_{1}f_{6}+a_{2}f_{6}^{3}+a_{3}f_{6}^{5}=a_{1}y_{1}+a_{2}y_{1}^{3}+a_{3}y_{1}^{5}\\ \begin{array}{cc} f_{10}=\frac{1}{R_{Z}}e_{10}=\frac{1}{R_{Z}}u, & f_{11}=C_{P}\frac{de_{11}}{dt}=C_{P}\frac{du}{dt}. \end{array} \end{array}$$

The last component of the bond graph, which has not been described so far, is the energy transforming element “TF”, whose task is to convert mechanical energy into electrical energy. Appropriate analytical relationships, taking into account the cause-and-effect relationships of the incident edges with the transformer, take the following form:

(4) $$\begin{array}{cc} e_{8}=k_{P}e_{9}=k_{P}u, & f_{9}=k_{P}f_{8}=k_{P}{\dot{y}}_{1} \end{array}$$

The equation modeling the dynamics of the mechanical subsystem was obtained directly as a result of substituting Equation (3) to Equation (1):

(5) $$\begin{array}{cc} e_{6}+e_{7}+e_{8}-e_{5}=0, & e_{3}+e_{4}-e_{2}=0,\\ ↓ & ↓\\ e_{6}-e_{5}+e_{7}+e_{8}=0, & e_{5}=e_{2}=e_{3}+e_{4},\\ ↘ & ↙ \end{array}\;\begin{array}{c} e_{6}-e_{3}-e_{4}+e_{7}+e_{8}=0,\\ ↓\\ m_{1}\frac{d{\dot{y}}_{1}}{dt}-b_{B}\left({\dot{y}}_{0}-{\dot{y}}_{1}\right)-c_{B}\int \left({\dot{y}}_{0}-{\dot{y}}_{1}\right)dt+a_{1}y_{1}+a_{2}y_{1}^{3}+a_{3}y_{1}^{5}+k_{P}u=0. \end{array}$$

The equation responsible for the dynamics of the electrical subsystem was obtained from the relationships corresponding to the zero nodes, as follows:

(6) $$\begin{array}{c} f_{11}+f_{10}-f_{9}=0\\ ↓\\ C_{P}\frac{du}{dt}+\frac{1}{R_{Z}}u-k_{P}{\dot{y}}_{1}=0 \end{array}$$

Comparing the derived differential equations of the second order (5) and the first order (6), a generalized electromechanical mathematical model of the tested design solutions of energy harvesting systems was obtained. Its dimensional representation is presented by the system of equations in (7):

(7) $$\left\{\begin{array}{l} m_{1}\frac{d^{2}y_{1}}{dt^{2}}+b_{B}\frac{dy_{1}}{dt}+a_{3}y_{1}^{5}+a_{2}y_{1}^{3}+\left(c_{B}+a_{1}\right)y_{1}+k_{P}u=b_{y}\frac{dy_{0}}{dt}+c_{y}y_{0},\\ C_{P}\frac{du}{dt}+\frac{1}{R_{Z}}u-k_{P}\frac{dy_{1}}{dt}=0. \end{array}\right.$$

Further, the derived system of differential equations was transformed into a dimensionless form since such a representation of the mathematical model significantly affects the effectiveness of computer simulations. In addition, it was assumed that the tested design solutions of energy harvesting systems are affected by external force excitation with two (spring and damping) components, $b_{y}\frac{dy_{0}}{dt}+c_{y}y_{0}$, where *y*_0_ = *A*sin(*ωt*), where *A* and *ω* are the amplitude and frequency of excitation, respectively. The dimensionless equation becomes:

(8) $$\left\{\begin{array}{l} \ddot{x}+\delta \dot{x}+\left(\beta x^{4}+\alpha x^{2}+\left(1+\mu \right)\right)x+\theta u=h\omega p\cos\left(\omega \tau \right)+p\sin\left(\omega \tau \right)\\ \dot{u}+\sigma u-\vartheta \dot{x}=0 \end{array}\right.$$

where the corresponding parameters are defined as:

$$\begin{array}{ccccccc} \omega_{0}^{2}=\frac{c_{B}}{m_{1}}=\frac{c_{y}}{m_{1}}, & \tau =\omega_{0}t, & \omega =\frac{\omega_{W}}{\omega_{0}}, & p=\frac{A}{x_{0}}, & \mu =\frac{a_{1}}{c_{B}}, & x=\frac{y_{1}}{x_{0}}, & h=\frac{b_{B}}{m_{1}}=\frac{b_{y}}{m_{1}}, \end{array}\;\begin{array}{ccc} \delta =\frac{h}{\omega_{0}}, & \theta =\frac{k_{P}}{x_{0}m_{1}\omega_{0}^{2}}, & \begin{array}{cccc} \sigma =\frac{1}{C_{P}R_{Z}\omega_{0}}, & \vartheta =\frac{k_{P}x_{0}}{C_{P}}, & \beta =\frac{a_{3}x_{0}^{4}}{c_{B}}, & \alpha =\frac{a_{2}x_{0}^{2}}{c_{B}}. \end{array} \end{array}$$

The numerical values of the physical and geometrical parameters of the considered construction solutions, which formed the formal basis for conducting the numerical calculations, are listed in Table 1. For a comparison of the BEH and TEH system responses, we used dimensional representation of *u* (V).

**Figure 4:**
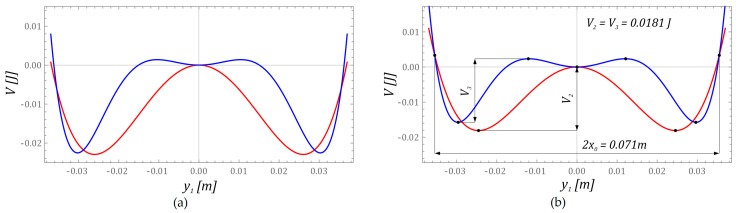
Potential characteristics of energy harvesting systems caused by: (**a**) magnetic field of distributed permanent magnets (Figure 1), and (**b**) magnetic field effect as in the previous case and an additional change in the stiffness of the flexible cantilever beam (to satisfy equal potential barriers, *V*_2_ = *V*_3_) used in further calculations.

At this point, it should be noted that the value of the scaling parameter *x*_0_ was adopted as the intersection point of the external potential barriers of the characteristics mapped with two and three wells (Figure 4b).

During the numerical calculations, the same mechanical properties of the flexible cantilever beam *I*, piezoelectric transducer *II*, and inertial elements loading the free ends of the beam were assumed same. In addition, in computer simulations, the depths of external wells were assumed the same, which were adjusted to the fourth decimal place. In addition, the potentials of the systems were selected in such a way that the distances measured between the extreme barriers at the level of *V* ≈ 0.003 J had comparable values. Adopting such model assumptions will enable a direct comparison of energy harvesting efficiency determined by model tests.

## 3. Results of Model Studies of Dynamic Properties

Based on the adopted model assumptions and numerical values to characterize the tested design solutions of energy harvesting systems, numerical calculations were carried out, the results of which are presented in the form of multi-color maps of the distribution of the largest Lyapunov exponent. This index is one of the key numerical tools applicable in the study of nonlinear dynamical systems. Its primary application is the identification of areas where unpredictable behavior of the dynamic system takes place. In particular, on its basis, the rate of separation of initially infinitely close trajectories on the phase plane was estimated. From the theoretical point of view, the largest Lyapunov exponent can be calculated using strict analytical and numerical methods [39,40,41]. In general terms, the method of identifying the largest Lyapunov exponent boils down to averaging over many iterations in an adequate phase space. In the formulated general model of the energy harvesting system, the phase space is defined by the displacement and velocity of the free end of the flexible cantilever beam $\left(x,\dot{x}\right)$. During model tests, the numerical procedure proposed by Wolf et al. [42] is most often used, as follows:

(9) $$\lambda =\underset{e\left(0\right)\to 0,n\to \infty }{\lim}\frac{1}{n\;\tau }\sum_{i=1}^{n}ln\left(\frac{\varepsilon_{i}\left(\tau \right)}{\varepsilon \left(0\right)}\right)$$

**Figure 5:**
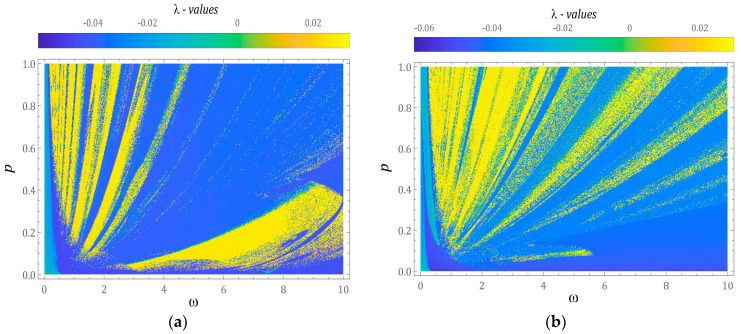
Multicolor maps of distribution of the largest Lyapunov exponent identified for systems with: (**a**) 2-well potential and (**b**) 3-well potential for the assumed zero initial conditions. Yellow regions correspond to chaotic solutions. *ω* and *p* are dimensionless.

In Equation (9), *ε_i_* (*τ*) represents the distance connecting, at the same instant of time, *τ*, the trajectory of the tested system with the reference trajectory. At the initial moment *τ* = 0, both trajectories are in close proximity. In our research, the distance between the beginnings of both phase streams was assumed to be *ε*(*τ* = 0) = 10^−5^. The results of numerical calculations, presented below, have been illustrated in the form of two-dimensional multicolored maps of distribution of the largest Lyapunov exponent (Figure 5).

Positive values of λ, represented by orange and yellow colors, indicate the chaotic behavior of the tested design solutions of energy harvesting systems. On the other hand, negative values of λ indicate that the phase trajectories are attracted by stable point attractors or periodic orbits. If λ takes values close to zero, then we are dealing with the so-called bifurcation points. At this point, it is noted that the results of computer simulations were obtained under the assumption of zero initial conditions. On the basis of the multicolored maps of the largest Lyapunov exponent, it can be concluded that in the case of a system whose potential is set via two wells (Figure 5a), in the range of low values of dimensionless amplitude, *p* < 0.4, mechanical vibrations affecting the energy harvesting system are located in the range of high frequencies, *ω* > 3. In the range of low values, *ω* < 3, responses assuming the nature of unpredictable solutions dominate in the range of high values of *p* > 0.3. If the barrier initiated by permanent magnets is set with a three-well potential (Figure 5b), then chaotic solutions occur in the range of large values of *p* > 0.5. At the same time, in the range of high excitation frequencies, the areas of chaotic solutions mix with the areas of periodic solutions. On the other hand, periodic responses are located in the range of low levels of dynamic interactions, *p* < 0.5.

**Figure 6:**
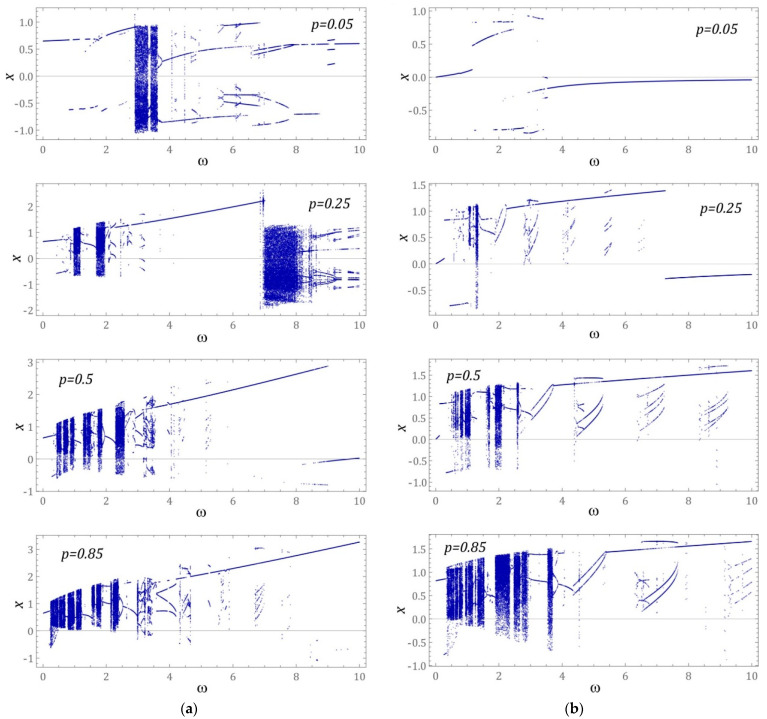
Bifurcation diagrams (stroboscopic) of steady states of the system against frequency for: (**a**) two potential wells and (**b**) three potential wells. The excitation amplitude increases downwards from 0.05 to 0.85 and its values are listed in the corresponding subfigures. The results were obtained for zero initial conditions. *ω* and *x* are dimensionless.

With regard to the selected values of the dimensionless amplitude of mechanical vibrations affecting the tested system, Feigenbaum steady-state bifurcation diagrams were generated (Figure 6). On their basis, it is possible to determine, among others, the nature of the solution and, as is the case with the largest Lyapunov exponent, the location of the zones of chaotic solutions. From a theoretical point of view, bifurcation diagrams can be generated in several ways. One of the most popular methods is based on local maxima and minima of time sequences of generalized coordinates of a mathematical model. Exactly the same results are obtained if the points of the steady-state diagram are identified by the intersections of the phase flow with the axis of the abscissa of the phase plane. The obtained graphic images of bifurcation diagrams are the same because they map the same values identified from different solution representations. At this point, it is worth mentioning that both approaches are characterized by the simplicity of numerical calculations, however, it is not always possible to precisely determine the periodicity of the solution through them. An example of a solution illustrating an incorrectly identified periodicity is shown in the orbits on the graphs (Figure 8a, *ω* = 6.5 and Figure 8b, *ω* = 8.75). For this reason, in our study, an alternative approach was used to identify steady states based on the intersection points of the phase flow with the control plane of the Poincare section. By using this approach, we are able to precisely determine the periodicity of the solution in relation to the given values of the control parameter. In our numerical calculations, the control parameter is the dimensionless frequency of mechanical vibrations affecting the tested design solutions of energy harvesting systems.

In the case of the energy harvesting system, the potential of which is given by two wells (Figure 6a), in the range of low values of the dimensionless amplitude of the external load *p* = 0.05, we are dealing with the dominance of periodic solutions. In the band *ω* < 3, the response of the system is a periodic solution with a periodicity of 1*T*. Solutions with such periodicity also occur in the range of very high frequencies, *ω* = 10. Responses with higher periodicity occur in the band *ω* ∊ [3.5, 9.25], with the vast majority of these solutions being 2*T*-periodic. The largest area of results, whose periodicity *T* > 2*T*, is located in the band *ω* ∊ [5.5, 8]. Chaotic responses are in the zone *ω* ∊ [3, 3.7]. In fact, these are two bands of unpredictable solutions located in very close proximity. Increasing the level of the external load to the value of *p* = 0.25 shifts the zones of chaotic solutions towards low values of *ω*. At the same time, in relation to the discussed case, a significant spreading of bands of unpredictable solutions was observed. It is worth noting that responses of this nature were induced in the high-frequency range, *ω* ∊ [7,8]. In the middle part of the bifurcation diagram *ω* ∊ [3.5, 7], periodic solutions with periodicity *T* > 1*T* were extinguished and replaced by 1*T*-periodic solutions, which were characterized by a large orbit of vibrations. A further increase of the dimensionless amplitude to the level of *p* = 0.5 caused bifurcations of the areas of chaotic solutions, which are located in the zone *ω* ∊ [0.5, 3.5]. In the range of high forced frequencies, *ω* > 5, the bifurcation diagram was dominated by high-energy periodic solutions with a periodicity of 1*T*. For *p* = 0.85, in the band *ω* ∊ [0.25, 2.5], there were basically homogeneous zones of chaotic responses. Periodic orbits with higher periodicities are located in the middle part of the generated bifurcation diagram, and for very high excitation frequencies, *ω* > 5, we are dealing with large vibrations of the flexible cantilever beam with a periodicity of 1*T*.

If the potential of the energy harvesting system was given in the form of three wells (Figure 6), then for low amplitudes of mechanical vibrations *p* = 0.05, the identified bifurcation diagram was dominated by periodic solutions with a periodicity of 1*T*. Responses with a higher periodicity were rare and most often occurred in the band *ω* ∊ [1.7, 3.5]. By increasing the level of external mechanical vibrations to the value of *p* = 0.25, it excited areas of chaotic and periodic solutions with a periodicity of 3*T* and higher. At the same time, for the external load determined in this way, the bands of occurrence of unpredictable responses were relatively narrow and fell within the variability range *ω* ∊ [1, 1.5]. A further increase in the amplitude of the external load to the level of *p* = 0.5 and *p* = 0.85 caused the bifurcation of chaotic solutions in the range of low frequencies, *ω* < 3 (*p* = 0.5) and *ω* < 5 (*p* = 0.85). In addition, periodic responses of systems with periodicities of 3*T* and higher were shifted towards high values of *ω*.

The results of computer simulations provided general information on dynamic properties. Based on them, it was not possible to conclude on the effectiveness of the tested design solutions of energy harvesting systems, because the points appearing in the bifurcation diagrams characterized the location of the intersection of the phase flow with the control plane of the Poincare section. Evaluation of the efficiency of energy harvesting, possible at the time of conducting supplementary numerical calculations, will be the subject of research in Section 4.

### 3.1. Dynamic Properties of a Two-Well System

**Figure 7:**
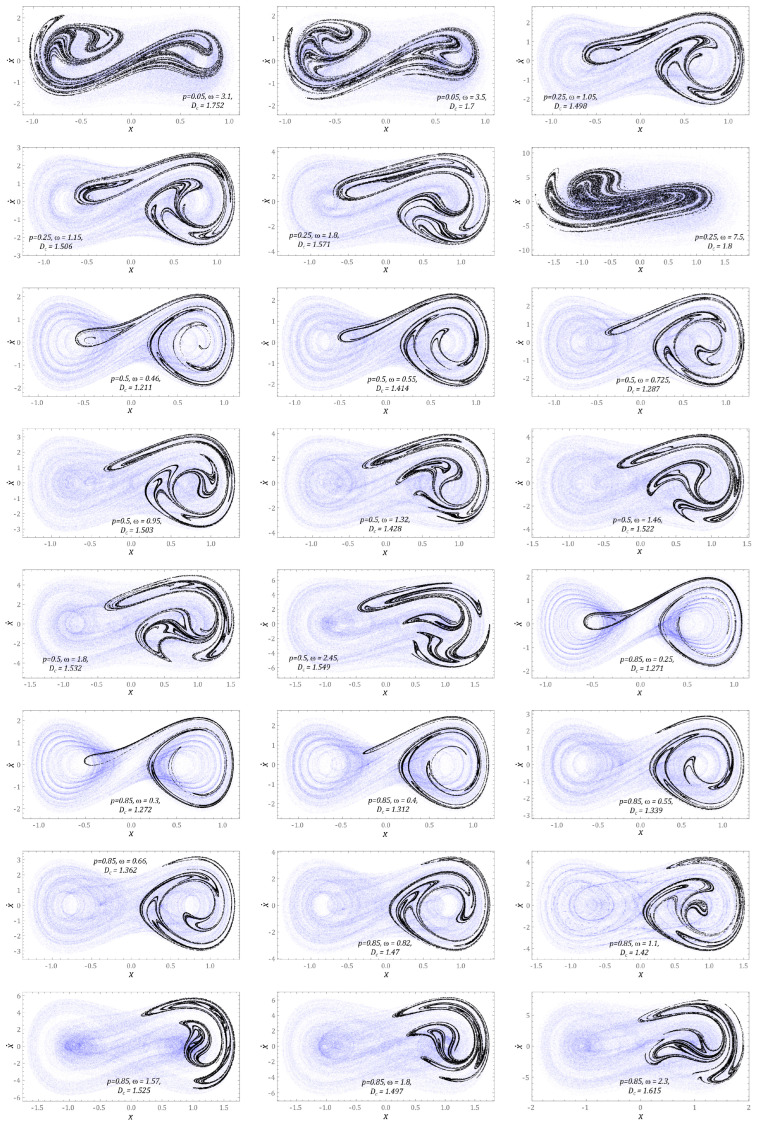
Exemplary solutions showing the geometrical structures of chaotic phase flows and the corresponding Poincare cross-sections of a BEH. *D_C_* denotes the corresponding correlation dimension. *ω*, *p*, *x*, and $\dot{x}$ are dimensionless.

This section presents the results of computer simulations, illustrating the dynamic properties of the energy harvesting system in which the potential characteristics were mapped with two wells. The scope of the model tests was limited to cases corresponding to zero initial conditions, because they correspond to the rest position of the flexible cantilever beam. The graphs (Figure 7) show the influence of dynamic load characteristics on the evolution of chaotic attractors.

At this point, it is noted that all graphical images included in the graphs (Figure 7) are plotted for the middle value of dimensionless frequencies, in bands of chaotic solutions. Chaotic attractors are depicted against the background of a phase flow, with the phase trajectory in the examined time interval represented by approximately 160 × 10^3^ points, which were additionally set to transparency. As a result of this approach, it was possible to observe the areas of the phase plane that are most often “visited” by the trajectory. This behavior of the phase stream was not observable when plotted with a solid line, because the use of a solid line tends to blur the trajectory image being plotted. The representation of the phase flow, in the form of a scatter plot, provided qualitatively new information about the phase flow by relating it to the *D_C_* correlation dimension of the Poincare section. We note that for chaotic attractors, whose correlation dimensions take *D_C_* > 1.5, the points representing the phase trajectory formed a fuzzy cloud. Such images of the chaotic phase flow occurred in the range of low values of the dimensionless excitation amplitude, *p* = 0.05. The highest value of the correlation dimension *D_C_* = 1.8 was recorded for the case *p* = 0.25 and the excitation frequency *ω* = 7.5. In this case, we are dealing with a “fat” Poincare section of the chaotic attractor, where the points of the digitized phase flow are arranged irregularly, or even randomly, on the phase plane. As the value of the correlation dimension decreased, the points representing the discretized trajectory were grouped into “rings”, potential barriers surrounding the wells. We recorded such geometrical structures of the phase stream of the correlation dimension assuming relatively small values of *D_C_* ≈ 1.2.

It is also worth noting that in the case of small excitations, the Poincare sections of the chaotic attractors were located in the vicinity of both wells of the potential barrier. Along with the increase in the value of the dimensionless amplitude of mechanical vibrations, *p*, affecting the two-well energy harvesting system, the chaotic attractors were attracted to one of the wells. In all the cases we examined, the Poincare sections of the chaotic attractors were attracted to the right potential well.

**Figure 8:**
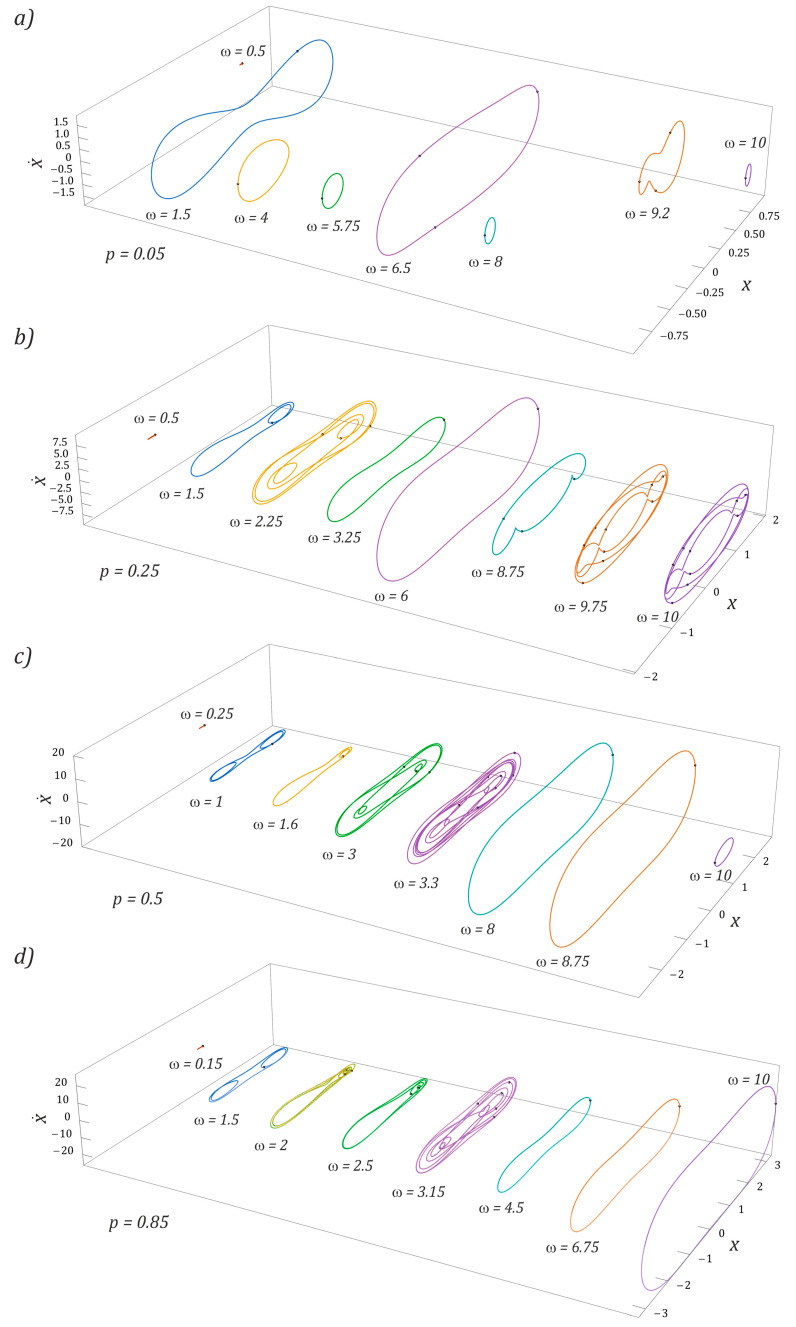
Examples of periodic responses of a BEH system identified for dimensionless mechanical vibration amplitudes: (**a**) *p* = 0.05, (**b**) *p* = 0.25, (**c**) *p* = 0.5, and (**d**) *p* = 0.85 (see the shape and size of the corresponding phase portrait $\left(x,\dot{x}\right)$). *ω*, *p*, *x*, and $\dot{x}$ are dimensionless.

The results of model tests indicate that the highest efficiency of energy harvesting was achieved by nonlinear dynamic systems when we dealt with large trajectories with periodic solutions [13,14]. For this reason, the orbits of stable periodic responses are presented in the rest of the paper. If the two-day energy harvesting system was subjected to an external dynamic load with an amplitude of *p* = 0.05 (Figure 8a), then stable periodic solutions with large orbits occurred in the vicinity of frequencies *ω* = 1.5 and *ω* = 6.5. In the first case, we are dealing with a response with a periodicity of 1*T*, while in the second situation, the response of the system is given a 3*T*-periodic orbit. In other cases, the solution orbits are located inside the potential well. For the external excitation, interacting with the amplitude *p* = 0.25 (Figure 8b), the trajectories of periodic orbits run around both potential wells. It is worth noting that only in the low-frequency range were we dealing with an asymmetric orbit, and the other examples correspond to symmetrical trajectories. As in the example (Figure 8a), the highest efficiency of energy harvesting was characterized by a solution represented by a large orbit, *ω* = 6. The results of model tests confirming this state of affairs will be presented later in this paper.

With regard to large amplitudes of the external dynamic load *p* ≥ 0.5, the vibration amplitude of the flexible cantilever beam increased with the increase in the dimensionless frequency value. Basically, in the entire range of variability, *ω*, we were dealing with orbits circling both potential wells. Only in the range of low values, *ω* < 0.3, and for very large values, *ω* ≥ 10, were there solutions whose trajectories are located inside the potential well (Figure 8c). The results of numerical calculations are shown in the diagrams (Figure 8d) and confirmed the thesis about the shift of the zones of periodic and chaotic solutions towards higher values of the dimensionless excitation frequency.

Regardless of the level of external load affecting the tested design solution of the energy harvesting system, in the low-frequency range, *ω* < 0.5, we dealt with solutions whose orbits are located inside the well of the potential barrier. These solutions showed a low energy harvesting efficiency, as a consequence of which the ability to harvest energy was limited. In the case of asymmetric orbits and those located inside wells, there was a high probability of multiple solutions.

### 3.2. Dynamic Properties of a Three-Well System

In the case of the design solution of the energy harvesting system, in which the potential is set via three wells, chaotic solutions occurred much less frequently (Figure 6b) in relation to the system with a two-well potential (Figure 6a). In addition, the bands of chaotic solutions were characterized by a much narrower band of their occurrence, and their permanent responses were induced in the range of higher loads affecting the tested *p* ≥ 0.25. It is worth noting that in relation to the system with a two-well potential, a smaller variety of identified Poincare cross-sections was also observed.

**Figure 9:**
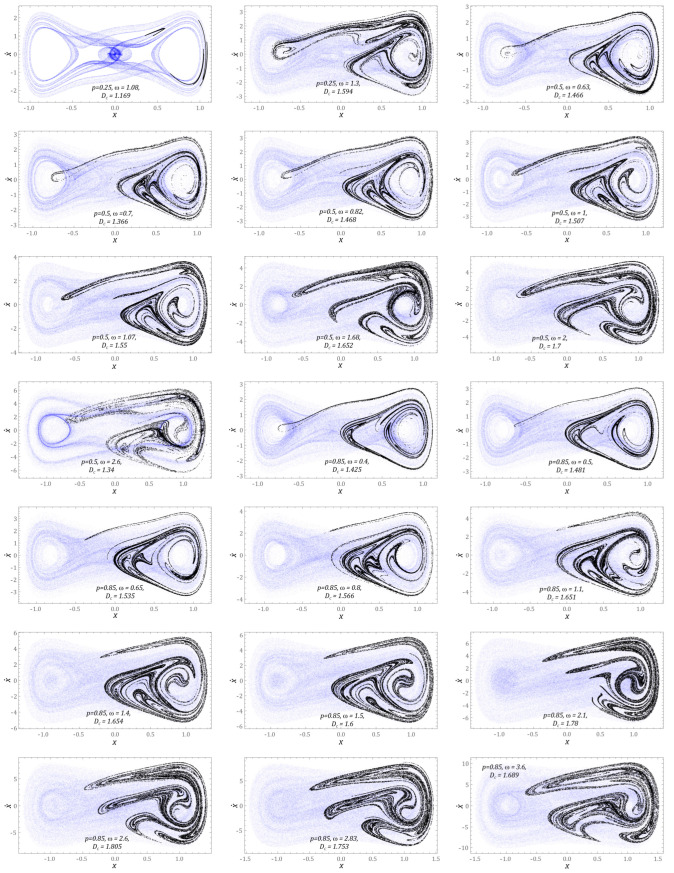
Example solutions showing geometric structures of chaotic phase flows and corresponding Poincare cross-sections, which were identified for a system with three potential wells (TEH). *D_C_* denotes the corresponding correlation dimension. *ω*, *p*, *x*, and $\dot{x}$ are dimensionless.

Analogous model tests were carried out for the energy harvesting system with three potential wells (Figure 9). For chaotic solutions, a similar behavior was observed as in the case of the two-well system. With the increase of the dimensionless amplitude of the external dynamic load acting on the energy harvesting system, the geometric structure of the chaotic attractor was attracted to one of the external potential wells. For low external load amplitudes of *p* = 0.05, no areas of chaotic solutions were recorded. The first signs of inducing responses of this nature appeared at *p* = 0.25. In this case, for *ω* = 1.08, we were dealing with three simultaneously coexisting chaotic attractors whose geometric structures are arranged on the phase plane along the path marked in blue. This solution is very similar to the quasi-periodic response, if only the area of phase space most often “visited” by the phase stream is taken into account. Such a statement is justified because the correlation dimension of the identified Poincare cross-section has values close to unity, *D_C_* = 1.169. For the structural solution in which the permanent magnets establish a three-hundred-day potential, as in the analyzed case, as the value of the correlation dimension of the Poincare cross-section increased, *D_C_* > 1.5, the points representing the phase flow became blurred, dynamic *p* ≥ 0.85. It is worth noting that the *D_C_* level was significantly higher in relation to the system based on two potential wells.

**Figure 10:**
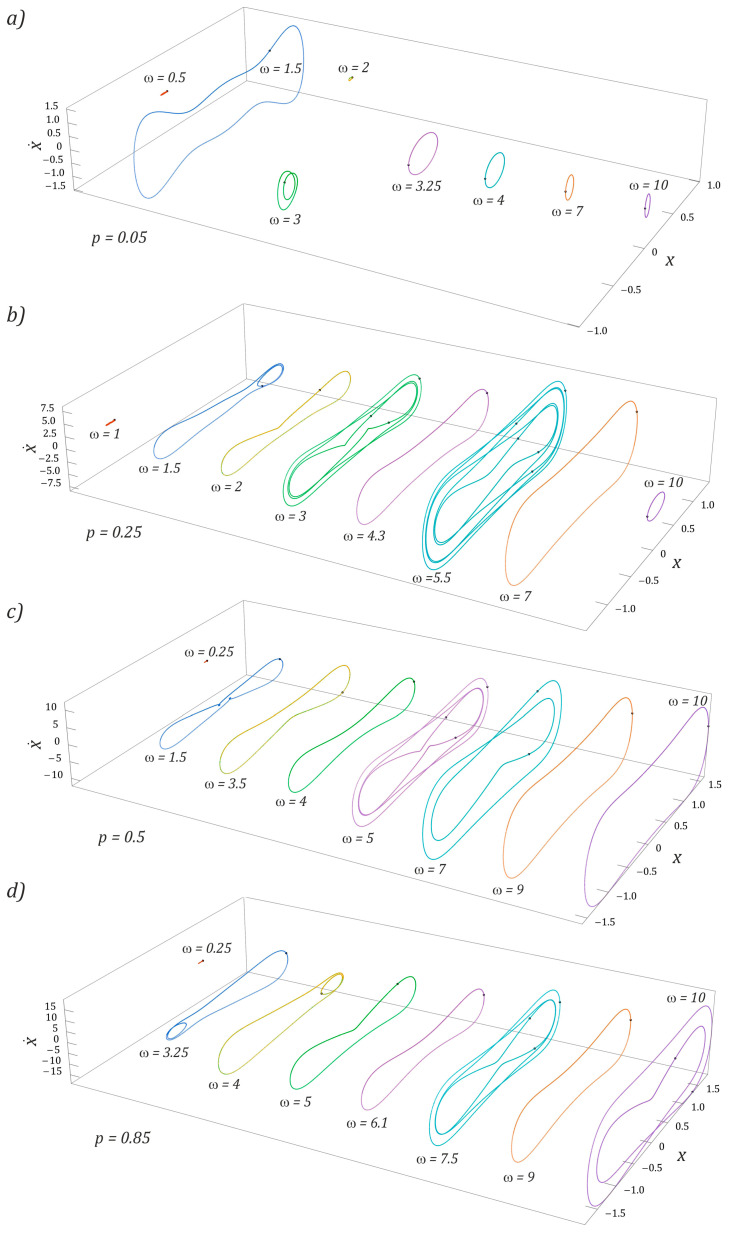
Influence of external load characteristics on periodic induced solutions (see the shape and size of the corresponding phase portrait $\left(x,\dot{x}\right)$) in a TEH. (**a**) *p* = 0.05, (**b**) *p* = 0.25, (**c**) *p* = 0.5, and (**d**) *p* = 0.85. Trajectory shapes are plotted for selected frequencies, *ω*. *ω*, *p*, *x*, and $\dot{x}$ are dimensionless.

The evolution of stable periodic solutions, which are excited in individual cross-sections of the multicolored distribution map of the largest Lyapunov exponent (Figure 5b), is shown in the graphs in Figure 10.

In the range of low values of the dimensionless amplitude of the external load affecting the energy harvesting system with the three-well potential, a stable periodic solution characterized by a large orbit occurred in the range of low values, *ω* < 2 (Figure 10a). In the remaining variability bands, *ω*, we were basically dealing with solutions characterized by low efficiency of energy harvesting. This is because their orbits are limited by local potential barriers. With the increase in the amplitude of the external load (Figure 10b), solutions whose orbits circulate around the external potential wells were excited, as a result of which the efficiency of energy harvesting from mechanically vibrating objects was significantly improved. The initiated periodic responses were characterized by low periodicity, the highest value of which, *5T*, was observed for *ω* = 5.5 (Figure 10b). A further increase in the value of the amplitude of mechanical vibrations of *p* ≥ 0.5, affecting the tested design solution, had a positive effect on the efficiency of energy harvesting, because solutions represented by large orbits were released.

It is worth noting that, regardless of the frequency and amplitude of the external load acting on the system with the three-well potential, basically all excited periodic solutions were characterized by odd periodicity. Only in the case of orbits *ω* = 7 (Figure 10c) and *ω* = 10 (Figure 10d) were we dealing with a 2*T*-periodic solution, characterized by a large vibration amplitude of the flexible cantilever beam.

## 4. Impact of the Potential Barrier on Harvesting Efficiency

**Figure 11:**
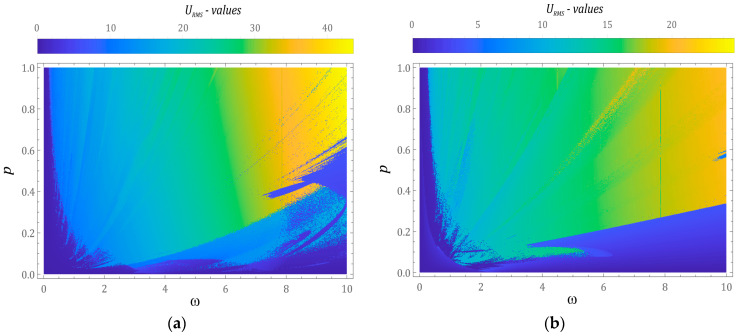
Multicolored maps of the values of effective energy harvesting systems (RMS voltage outputs) with the (**a**) two-well (BEH) and (**b**) three-well (TEH) potential for zero initial conditions. *ω* and *p* are dimensionless, while *U_RMS_* is expressed in Volts.

The effectiveness of the energy harvesting system mainly depends on the efficiency of energy acquisition. There are many qualitative indicators to assess this. The most popular one of them, and at the same time the simplest to use, is based on the RMS values of electric power and voltage induced on the piezoelectric electrodes. In our research, the measure of effectiveness was the effective value of the voltage induced in the system with the two-well and three-well potential. According to the authors, the use of such an indicator provides directly qualitative and quantitative information about the tested design solutions of the energy harvesting system. The results of numerical calculations, showing the ability to recover energy in a wide range of variability of control parameters, are presented in the form of multi-colored maps of the distribution of effective values of systems with a two-well and three-well potential (Figure 11).

**Figure 12:**
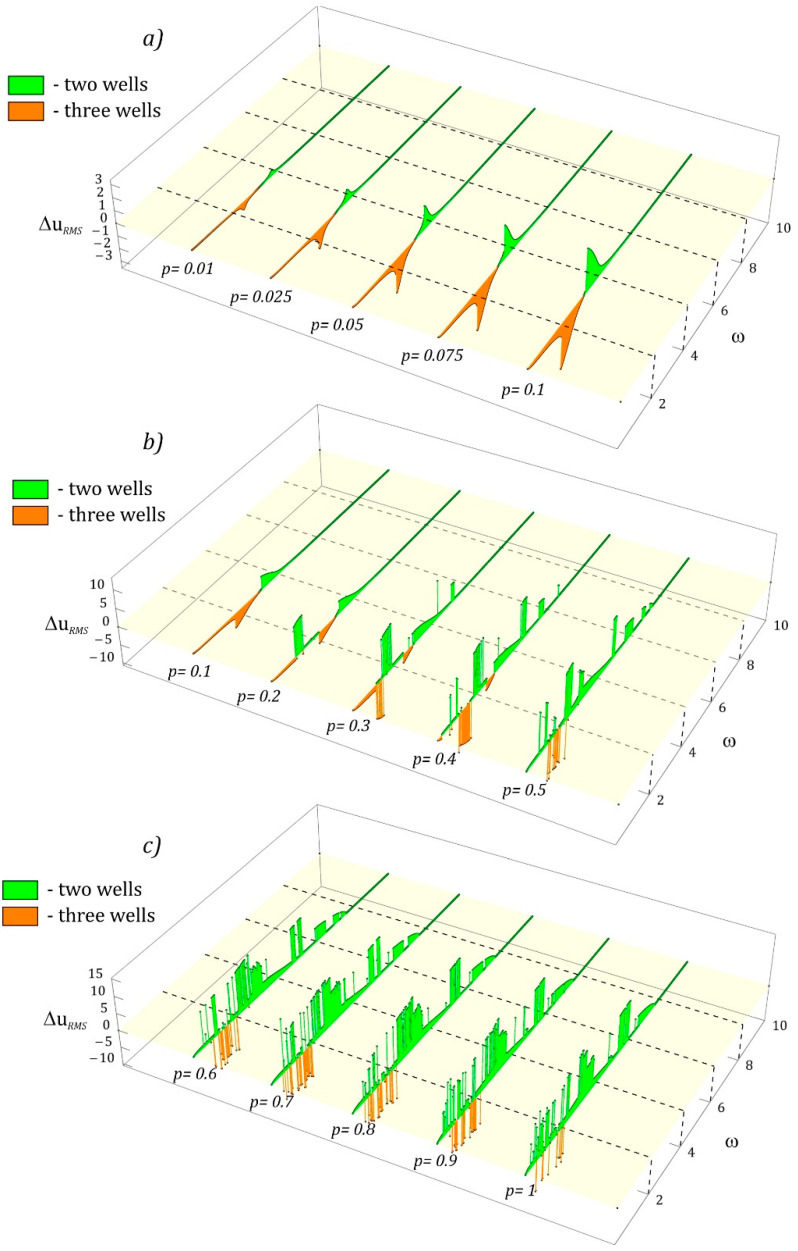
Comparison of RMS voltage outputs for two- and three-well potential systems versus amplitude and frequency. *ω* is dimensionless, while *U_RMS_* is expressed in Volts.

Irrespective of the tested construction solution, the identified multicolored maps were characterized by a similar geometric structure of the distribution. The highest efficiency of energy harvesting, in both cases, occurred for longer amplitudes and frequencies of mechanical vibrations affecting energy harvesting systems. Nevertheless, in the case of a system with a two-well potential, we were dealing with almost twice as high RMS maximum values. The results of numerical simulations, which directly compared the energy harvesting capacity, in relation to selected values of the dimensionless amplitude of the external load (Figure 12), are presented below.

A direct comparison of both construction solutions was possible, because the geometrical and material dimensions characterizing the flexible cantilever beams were the same. In addition, during the planning of numerical calculations, permanent magnets were placed, defining potential barriers in such a way as to ensure comparable potential widths and depths of external wells (Figure 4b).

Based on the results of numerical simulations, it can be concluded that in the entire analyzed range of external load frequency variability, it is possible to distinguish three characteristic bands. This is the case in the range of low amplitudes, *p* < 0.1 (Figure 12a). In the high band, *ω* > 5, systems with the two-well and three-well potential can be used interchangeably, because the identified differences in the RMS values of the voltages induced on the piezoelectric electrodes, in principle, did not show statistically significant differences. The situation is different in the transition band 3 < *ω* < 5, in which the system with a two-well potential showed a better efficiency of energy harvesting in relation to the system with a three-well potential. On the other hand, in the low-frequency zone, *ω* < 3, the design solution based on the three-well potential showed a better energy harvesting efficiency. The maximum differences in effective voltages in the considered range, *p* ≤ 0.1, did not exceed the value of 3 V.

With the increase in the level of mechanical vibrations affecting the tested construction solutions, the ability to harvest energy in the system with the two-well potential improved in relation to the system based on the potential barrier with three wells (Figure 12b). At the same time, in this considered range of external load amplitude variability, 0.1 < *p* < 0.5, one can refer to a statistically significant increase in the efficiency of energy harvesting, because the values of the effective voltage differences reached the level of approximately 10 V. However, in the range of high frequencies, *ω* > 8, two-well and three-well systems can still be used interchangeably, due to negligibly small differences in the voltages induced on the piezoelectric electrodes. A system with a three-well potential can effectively recover energy only in the range of low values, *ω* < 2. Nevertheless, such a situation occurs in relatively narrow bands of *ω* variability.

With regard to high vibration levels, *p* > 0.6, regardless of the frequency of the external load, it is recommended to use a design solution based on the two-well potential (Figure 12c). In fact, in the range of low *ω* < 2, there are areas where the system with the three-well potential showed better energy harvesting properties. Nevertheless, these are relatively narrow bands that can cause certain difficulties. At the same time, these difficulties are mainly caused by the need to tune the converter to the current load conditions affecting the energy harvesting system.

## 5. Conclusions

A variety of BEHs and TEHs were presented in previous scientific papers [11,12,13,16,27,30,31,32,33,43,44], which prompted a comparative analysis of these systems. We compared the efficiency of obtaining energy from such systems, by assuming similar potential parameters with the increasing amplitude of harmonic excitation. The ability of the energy harvesting system with two-well potential improved in relation to the potential system with three wells. In the range of high frequencies, two-well and three-well systems can be used interchangeably, due to negligibly small differences in the voltage outputs. A system with a three-well potential can effectively harvest energy only in the range of low-frequency values. Note that the complete comparison of the BEH and TEH systems should involve the systematic change of the barrier height, which has been left for the future analysis.

The impedance optimization of the system was not analyzed in this paper. In the studied nonlinear system, it would depend on the specific solution together with its response frequency, however the input and circuit impedances can be adjusted by means of active converters [45].

Note that the numerical calculations were performed for the zero initial conditions. To find all the solutions, it is important to explore a larger set of them by applying the random selection of initial conditions or systematically analyzing the corresponding basins of attraction. Laboratory experiments are also planned in the next step. The results of such extended investigations will be reported in a future paper.

## Figures and Tables

**Table 1:** Systems’ parameters.

Name	Symbol	Value	
		Two Wells	Three Wells
Mass loading of the beam	*m*	0.0065 kg	
Energy losses in a mechanical system	*b_B_*	0.05 Nsm^−1^	
Stiffness of the beam	*c_B_*	15 Nm^−1^	
Damper coupling of the excitation force	*b_y_*	0.05 Nsm^−1^	
Spring coupling of the excitation force	*c_y_*	15 Nm^−1^	
Resistance of the electrical circuit	*R_Z_*	1.1·10^6^ Ω	
Capacity of the electric circuit	*C_P_*	72 nF	
Electromechanical constant of piezoelectric	*k_P_*	3.985·10^−5^ Nm/V	
Displacement scaling parameter	*x*_0_	0.0355 m	
Load coefficients caused by the influence of the magnetic field	*a*_1_ *a*_2_ *a*_3_	−135 Nm^−1^ 198.8·10^3^ Nm^−3^	53.7 Nm^−1^ −554.38·10^3^ Nm^−3^ 5.4·10^8^ Nm^−5^

## Data Availability

Data are contained within the article.
